# RNA-Seq for Plant Pathogenic Bacteria

**DOI:** 10.3390/genes2040689

**Published:** 2011-10-13

**Authors:** Jeffrey A. Kimbrel, Yanming Di, Jason S. Cumbie, Jeff H. Chang

**Affiliations:** 1 Department of Botany and Plant Pathology, Oregon State University, Corvallis, OR 97331, USA; E-Mails: kimbreje@science.oregonstate.edu (J.A.K.); cumbiej@onid.orst.edu (J.S.C.); 2 Molecular and Cellular Biology Program, Oregon State University, Corvallis, OR 97331, USA; 3 Department of Statistics, Oregon State University, Corvallis, OR 97331, USA; E-Mail: diy@stat.oregonstate.edu; 4 Center for Genome Research and Biocomputing, Oregon State University, Corvallis, OR 97331, USA

**Keywords:** next generation sequencing, transcriptomics, differential gene expression, overdispersion, negative binomial

## Abstract

The throughput and single-base resolution of RNA-Sequencing (RNA-Seq) have contributed to a dramatic change in transcriptomic-based inquiries and resulted in many new insights into the complexities of bacterial transcriptomes. RNA-Seq could contribute to similar advances in our understanding of plant pathogenic bacteria but it is still a technology under development with limitations and unknowns that need to be considered. Here, we review some new developments for RNA-Seq and highlight recent findings for host-associated bacteria. We also discuss the technical and statistical challenges in the practical application of RNA-Seq for studying bacterial transcriptomes and describe some of the currently available solutions.

## 1. Introduction: A Sneak Peek into RNA-Seq

Genome sequences for host-associated bacteria are being generated at an extraordinary rate. Their availability has had important contributions towards deciphering the highly complex and fascinating biological interactions between symbionts and their hosts. Since the 2000s, when the first genome sequences of plant pathogens were determined, we have gained a greater appreciation into the mechanisms of virulence, such as secretion systems and repertoires of effectors, metabolic and biosynthetic capacities to adapt to different environments, biosynthesis of secondary metabolites and toxins to modulate host plants, and evolution as well as taxonomical relationships of plant pathogenic bacteria [1–9].

Genome sequences are by no means the end of the road. A genome sequence is a map with the challenge of exploration to improve and make sense of it. As of six years ago, even *Escherichia coli*, the most heavily studied bacterium, had only 54% of its genes experimentally supported with another 32% computationally predicted [10]. No plant pathogenic bacterium is close to this level, as isolates belonging to the *Pseudomonas*, *Xanthomonas*, *Ralstonia*, and *Agrobacterium* genera have between 27%∼37% of their genes annotated as “hypothetical”. Adding to the challenges of studying plant pathogens is the amount of redundancy coded in their genomes and the subsequent difficulties that experimental biologists face in their efforts to map and characterize genes necessary for virulence [1].

Transcriptomic-based approaches have the potential to help rapidly address this knowledge gap. A transcriptome represents all RNA molecules, including the coding mRNAs as well as the noncoding rRNA, tRNA, sRNAs, *etc.* Investigators have mostly focused on protein coding mRNAs and, more recently, on the regulatory small RNAs, while excluding the “housekeeping” functional RNAs, such as rRNA, and tRNAs. As such, from hereafter, we use “transcriptome” to imply only mRNAs and sRNAs. The transcriptome is dynamic and is constantly changing in response to endogenous and exogenous cues. Thus, transcriptomic-based approaches typically rely on the characterization of snapshots captured from cells subjected to conditions and times of interest.

Microarrays were one of the earliest tools that offered researchers the once unique opportunity to investigate the reprogramming of a phytopathogenic bacterium's entire transcriptome. Microarrays were used to identify virulence regulons and study the physiological changes that occur in response to plant signaling molecules or in conditions that mimic the host environment [4,11–16]. Microarrays have some constraints that cap the possible explorations into transcriptomes. Microarrays are designed according to an available genome sequence and may have limited use to only its corresponding isolate, or at best to a small number of genetically similar isolates. Additionally, microarrays are limited by the quality of the genome sequence and annotation. As a consequence, except for the genome tiling arrays, most microarrays cannot be used for gene discovery and refinement of genome annotations for improving future transcriptomic-based inquiries without subsequent redesigns.

Next generation (next gen) sequencing has pushed data generation into the logarithmic growth phase. Several next gen platforms are available that use different chemistries but offer the same advantages over traditional Sanger sequencing-dramatic increases in throughput with decreases in cost, time, and labor (reviewed in [17]). The application of next gen sequencing to transcriptomics has been coined the inaccurate term of RNA-Sequencing or RNA-Seq, which is, in practice, simply the highly parallelized sequencing of cDNA fragments. Direct sequencing of mRNA has also been demonstrated, but this approach has not yet been widely adopted [18]. As will be discussed, there are different preparation methods for RNA-Seq to yield different levels of information regarding the transcriptome.

RNA-Seq has been used for expression profiling as well as many other explorations into transcriptomes. Analysis of RNA-Seq has shown that, despite the perceived relative simplicity of bacterial genomes in comparison to their eukaryotic hosts, bacterial transcriptomes and their regulation are nonetheless similar in complexity. Genes that escaped annotation have been uncovered using RNA-Seq, the most prominent being those of noncoding or small RNAs [19–26]. Subsequent characterization of sRNAs will contribute to a more comprehensive understanding in transcriptome regulation, as sRNAs largely function in gene regulation (reviewed in [27]). Analysis of RNA-Seq data derived from cDNA fragments prepared using enzymatic modifications to distinguish sense versus anti-sense strands or preprocessed versus processed transcripts, have helped to resolve overlapping or embedded genes as well as disputed operons, and identify transcript isoforms originating from alternative start sites [21,24–26,28,29]. In general, transcriptional initiation within upstream coding regions, anti-sense expression, and presence of alternative transcriptional start sites appear to occur with much higher prevalence than originally thought for bacterial genomes.

A distinct advantage of RNA-Seq is that cDNA fragments are directly sequenced and the reads can be *de novo* assembled to study organisms with no available reference genome sequence [30,31]. For bacteria, a more cost-effective and practical alternative is to combine analysis of RNA-Seq data with a draft genome sequence derived from next gen sequencing. This approach was successfully used to provide sufficient insights into the metabolic demands of a leech symbiont for the development of media to enable its culturing [32]. Furthermore, because of the single-base resolution and the ability to computationally predetermine and filter out ambiguous reads, RNA-Seq can also be used to study co-inhabitant or co-cultured microbes without concern for issues such as the unknowable cross-hybridization associated with microarrays [32,33]. Thus, RNA-Seq could be used to study the potential synergistic or antagonistic interactions that occur in plant-pathogenic bacterial communities such as the case with the soft rot *Pectobacterium carotovora* [34].

On the surface, with these advantages, it almost seems absurd to not use RNA-Seq. Millions to billions of RNA-Seq reads, terabytes of data, will be available quickly and cheaply. However, to date, there has been only a single report describing the use of RNA-Seq to study the transcriptome of a plant pathogen, *Pseudomonas syringae* [25]. For many researchers, the outlook becomes bleak when faced with the task of handling and making sense of the massive amounts of data. Unlike analysis of microarrays, there are no out-of-the-box or one-size-fits-all packages for analysis of RNA-Seq for bacteria. Also, with RNA-Seq data, there may be concerns with computational hardware. Depending on the organism and scope of RNA-Seq experiment, a desktop computer is most likely insufficient.

RNA-Seq, its uses and its analytical tools, are still in their developmental stages. In the following, we briefly review options for preparing RNA from bacteria as well as some of the computational challenges associated with RNA-Seq. Many of these topics have been comprehensively reviewed [17,35–38]. We then turn our attention to the statistical challenges of analyzing RNA-Seq data, with emphasis on analysis of differential gene expression.

## 2. Techniques for RNA-Seq Preparations

One of the first tasks of RNA-Seq is to produce a transcriptome depleted of rRNAs and tRNAs. These functional RNAs typically exceed 90% of the total RNA preparation and will likely represent >99% of the RNA-Seq reads if not sufficiently addressed [33]. In eukaryotes, mRNAs are processed in part by addition of a 5′ m7GpppX cap and 3′ poly(A) tail, which can be exploited to enrich for mRNAs. In prokaryotes, these features are not present. Rather, newly synthesized or preprocessed RNAs have a triphosphate at the 5′ end and the processed RNAs, such as rRNA and tRNAs, bear a 5′ monophosphate. As a consequence, many of the available methods for transcriptomes of bacteria deplete the unwanted RNAs from preparations.

For many experiments, the tRNAs and 5 s rRNA are of little concern because they can be excluded simply based on their small sizes. However, a fraction of the sRNAs may also be lost with these approaches as some sRNAs are as small as 50 nucleotides in length [39]. Thus, if one uses a preparation method to specifically capture smaller sized RNAs, an approach to deplete tRNAs and 5 s RNAs should be considered, otherwise only a small percentage of the reads will be informative [19].

**Figure 1:**
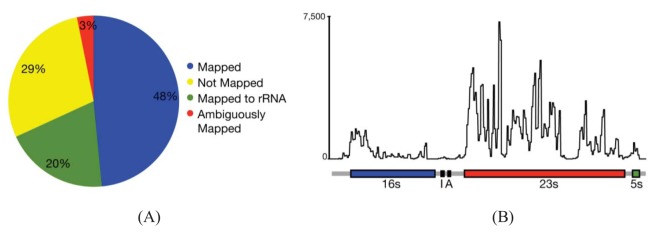
Categorization of RNA-Seq reads. (**A**) Alignment of 24,202,967 RNA-Seq reads to a *P. syringae* reference genome sequence. The rRNAs were depleted using Ribominus and MicrobExpress. The remaining RNA were converted to cDNA and sequenced on an Illumina IIG using single-direction 40-cycle sequencing. The first 10 and last five bases of each read were trimmed off. The 25 mers were pooled across six samples and aligned using the alignment program, CASHX version 2.3, allowing up to two mismatches. Reads were categorized based on alignment to a unique position (Mapped), the rRNA-encoding locus (Mapped to rRNA), failure to align (Not Mapped), and alignment to multiple locations in the reference genome sequence (Ambiguously Mapped). (**B**) Distribution and frequency of 25 mer RNA-Seq reads that aligned to the rRNA-encoding locus of *P. syringae* following rRNA-depletion. Reads were aligned using CASHX version 2.3.

In most cases, the concern is with the 16 s and 23 s rRNAs. Three methods are commercially available that address these abundant rRNAs. Subtractive hybridization is the most popular, e.g., MicrobExpress (Ambion, Austin, TX) and Ribominus ([40]; Invitrogen, Carlsbad, CA). Subtractive hybridization is straightforward and relies on bead-associated oligonucleotides complementary to 16 s and 23s sequences to deplete undesired rRNAs. One feature that distinguishes Ribominus from MicrobExpress is its use of locked-nucleic acids (LNAs) in the rRNA capture oligonucleotides [40]. LNAs are nucleotide analogs capable of complementary basepairing but with much higher thermal affinities allowing for the use of a higher temperature during depletion steps to increase the specificity of rRNA capture [41]. We have found that one round of MicrobExpress followed by a round of Ribominus is effective for removing a large fraction of the rRNA from RNA preparations of *P. syringae* (Figure 1A). Using qRT-PCR to assess efficiency of depletion, on average, less than 0.01% and 10% of the 16 s and 23 s rRNA, respectively, remained relative to the starting preparation (Kimbrel and Chang, unpublished). After sequencing, on average, approximately 20% of the reads aligned to the rRNA-encoding locus with 17% and 83% of those corresponding to the 16 s and 23 s rRNA, respectively. In our best case, only 12% of the total RNA-Seq reads corresponded to rRNA.

Since subtractive hybridization is a method of depletion, one must resist the temptation to use more input RNA than recommended, otherwise the transcriptome preparation may not be sufficiently devoid of rRNAs. Additionally, one needs to consult the list of compatible bacteria to determine whether the commercially available capture oligonucleotides will work for one's bacterium of interest. If inadequate, species-specific capture oligonucleotides can be designed but researchers should be aware that, due to post-transcriptional processing of precursor rRNA, the molecule is often fragmented and can exist as multiple, separate fragments [42]. Oligonucleotides should therefore be designed to several locations along the 16 s and 23 s-encoding rRNA to sufficiently capture each of the processed forms. Processing may contribute in part to the peaks and valleys pattern of RNA-Seq read alignment to the rRNA-encoding locus (Figure 1B).

The processed rRNAs can also be preferentially degraded using a 5′-Phosphate-Dependent Exonuclease (Terminator; Epicentre, Madison, WI). This approach has important implications in downstream data analyses and can be used to characterize bacterial transcriptomes with greater precision (see below). A third and relatively new method uses enrichment by relying on “not so random” oligonucleotides during cDNA preparation to bias towards non-rRNA transcripts [43] (Ovation^®^ Prokaryotic RNA-Seq System; NuGen, San Carlos, CA [44]). Finally, one last method is to simply sequence all cDNA fragments and computationally filter out reads corresponding to rRNA [26,33]. This method may have its appeal because there are no upfront investments of labor or cost to address rRNAs and no biases associated with the rRNA depletion methods. With the depth that can be achieved nowadays, throwing away 99.9% of the reads may still yield a substantial number of reads. Nevertheless, there is a considerable risk that if the necessary depth of sequencing is not obtained, there will be an insufficient number of informative reads for hypothesis generation or testing. Additionally, post-RNA-Seq filtering is not the most cost-effective method because the need to achieve sufficient depth of sequencing likely precludes the use of multiplex sequencing (see below).

In addition to rRNAs, we have also found that a tmRNA can sometimes be very abundant [45]. tmRNA is a bifunctional RNA that acts as both a tRNA and an mRNA in a process called trans-translation (reviewed in [46]). The high representation of tmRNA by RNA-Seq reads makes this gene a candidate worth considering for depletion prior to sequencing. Alternatively, it may be a candidate for post-RNA-Seq filtering. Its extremely high level of expression, relative to non-rRNA-encoding genes, has the potential to upset statistical testing of differential expression.

There are several methods to consider for preparing RNA for sequencing. The most straightforward method relies on sequencing randomly primed cDNAs and is sufficient for discovering genes, improving genome annotations, and assessing the transcriptome for gene expression changes. Strand-specific sequencing, in which the 3′ ends of transcripts are defined using a modification to the 3′ end prior to cDNA conversion, allows for a more precise interrogation of the transcriptome by distinguishing genes that are overlapping and expressed from different strands. Finally, treatment of RNA with a 5'-Phosphate-Dependent Exonuclease can be used to enrich preprocessed transcripts, which can help resolve alternative transcriptional start positions as well as overlapping and/or nested genes. Sharma *et al.*, for example, developed a method they called differential RNA-Seq in which two different preparation methods were used to process fractions of RNA derived from the same sample to distinguish strand-specific 5′ preprocessed transcripts [24]. Transcriptional start sites were then determined based on an enrichment of reads from the processed fractions relative to the unprocessed fractions. Operons were also inferred in combination with bioinformatic predictions and strand-specific sequencing. This approach has provided the most detailed view into the transcriptome of a bacterium so far.

Sharma *et al.*, did not fragment or size-select the RNA molecules prior to conversion to cDNA [24]. These steps are common to many RNA processing methods. Fragmentation has the potential to introduce some biases, such as sequence-specific effects on the efficiency of reverse transcription, adaptor ligation, or sequencing. Additionally, as described below, fragmentation has the potential to affect conclusions on differential expression in certain situations. However, skipping the fragmentation and size selection steps has some important considerations. First, this approach limits the sequencing platform that can be used since recommended fragment sizes for the Illumina, for example, are less than 650 bp. Furthermore, regardless of the sequencing platform, cDNAs of longer transcripts may be less represented because cDNA synthesis is done using oligonucleotides complementary to a 3′ adapter sequence. Products are further amplified to enrich for products and again to amplify fragments for sequencing. Each of these steps tends to favor shorter products. However, as described below, technical biases that affect all sample preparations similarly are not expected to have major effects on conclusions regarding differential expression.

**Figure 2:**
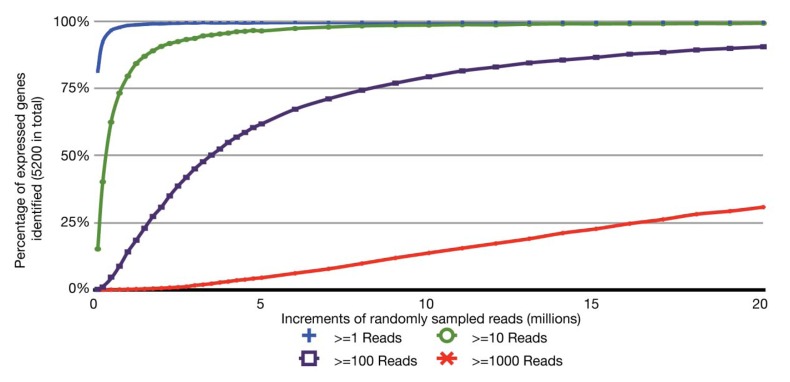
Identification of expressed protein-coding genes as a function of sequencing depth. Increments of reads (x-axis) were randomly sampled from the set of ∼24 million 25 mer reads (see Figure 1A) and aligned to a *P. syringae* reference genome features derived from the .ptt file (table of protein-coding features). The percent of expressed protein-coding genes discovered, relative to the ∼5,200 identified using all 24 million 25 mers, were plotted based on a minimum of 1 (blue), 10 (green), 100 (purple) or 1,000 (red) reads (y-axis).

With the relatively small transcriptome sizes of plant pathogenic bacteria, one can consider using bar coding of different sample preparations and multiplex sequencing to help reduce the cost of RNA-Seq experiments. Bar coding is the addition of nucleotide sequences that uniquely identify different sample preparations. Multiplex sequencing is simply the pooling of the bar-coded samples for more cost-effective simultaneous sequencing. A concern with this approach is the reduction in the average numbers of reads per gene and decrease in statistical power, *i.e.*, ability to identify truly differentially expressed genes. This is of greater concern with lowly expressed genes. The relation between sequencing depth and percentage of identified expressed genes for an RNA-Seq experiment of *P. syringae* is presented (Figure 2). With just ∼3.5 million pre-filtered reads, 95% of the annotated, expressed protein-coding genes are represented by at least 10 RNA-Seq reads, with an average of 190 reads per gene. On an Illumina HiSeq, 3.5 million reads is easily far less than 1/10 of the number of reads expected from a single channel. Ultimately, one has to balance the tradeoff between cost and depth of sequencing. Furthermore, one needs to consider that, as more samples are pooled, there is an increasing challenge in combining approximately equal ratios of cDNA preparations to achieve approximately similar depths of sequencing for all samples. One also needs to consider the barcode sequences. We have observed that some “home-made” barcode sequences dramatically reduced the number of informative reads [45]. Commercially available multiplex sequencing kits are available and likely use rigorously tested and optimized barcodes and barcode combinations.

## 3. Computer Geek for RNA-Seq

One of the first steps of RNA-Seq data analysis is often the alignment of reads to a reference genome sequence to identify expressed genes (Figure 1A). Many short read alignment programs have been developed and the challenges these programs have in processing RNA-Seq have been comprehensively reviewed [37]. Briefly, one of the important challenges is the assignment of ambiguous reads. These are reads with sequences that can align to more than one locus in the genome and, in the case of eukaryotes, to multiple transcript isoforms. Programs that exclude ambiguous reads will cause genes or transcripts to appear depressed in expression. In contrast, programs that include ambiguous reads have the potential for incorrect assignment, which will also affect detection of gene/transcript expression. An additional concern for transcriptomes of eukaryotes is alternative splicing. A fraction of the RNA-Seq reads will not align to a genome reference sequence because their sequences span yet-to-be discovered splice junctions. Programs with computational and statistical methods to predict transcript isoform structures and assign reads to isoforms have been developed but how they perform for analysis of prokaryotic transcriptomes is unknown.

While splicing is of little concern in the analysis of bacterial transcriptomes, the density of bacterial genomes and the overlapping and nested genes do incur similar challenges in causing ambiguities in the accurate assignment of reads to genes. Based on alignments of RNA-Seq reads to a reference genome sequence of *P. syringae*, only 3% of the reads were considered ambiguous (Figure 1A). However, this measure is based solely on genome location and does not consider reads that align to the same location encompassed by overlapping genes. Furthermore, our analyses do not take into consideration ambiguities resulting from initiation from alternate start sites. We therefore expect the percent of ambiguous RNA-Seq reads of bacteria to be higher than indicated. Fortunately, as described above, different cDNA preparations for bacteria can be used to help resolve ambiguities.

Data analysis, long-term data storage, and backup are points of concern as researchers increase the scale and scope of their RNA-Seq experiments and improvements in next gen sequencing technology yield more data with longer sequence reads. Of utmost importance is sufficient Random Access Memory (RAM) and processors. RAM acts as a very fast temporary storage space for programs that track large quantities of information. RAM is therefore critical because it directly affects the amount of data that can be analyzed per unit of time before access to the hard drive is required. Processes that rely on the latter are slower by many orders of magnitude.

Researchers may need access to large computing resources. In the absence of institutional infrastructures, cloud computing centers are cost-effective alternatives, e.g., iPlant Collaborative's Atmosphere [47]. A cloud is a computing service that provides access to processors, RAM, and disk space from multiple computers. The cloud handles the distribution of the collective resources to individual programs. The major advantage to cloud computing is their scalability in which users are able to specify the amount of RAM, disk space, and number of processors needed when requesting for such services. Some RNA-Seq pipelines have been developed to run on a cloud [48,49]. One potential drawback is that the users must operate within the constraints of the cloud infrastructure.

## 4. Statistical Analysis of RNA-Seq: Eke! It's Greek to Me

RNA-Seq has been used to profile gene expression changes of host-associated bacteria [20,50–53]. Comparisons to analysis of microarrays clearly highlighted the advantages in sensitivity and comprehensiveness of RNA-Seq [26]. We emphasize that, if one desires to generalize statistical conclusions from the samples to a population, one has to use independent biological replicates that are representative of the population. Some of the earlier uses of RNA-Seq relied on only technical replicates or unreplicated experiments so the conclusions only applied to the single sample from which the RNA-Seq experiments were based on.

For microarrays, one of the first steps in data analysis is normalization to correct for differences in intensities across microarrays [54]. RNA-Seq data are similar and require normalization to correct for differences in library sizes, which is the total numbers of reads for a sample. A standard approach is to use a measure of relative frequency, such as reads per million mapped reads. The use of relative frequency is not without its potential issues [55]. With a fixed library size (a sequencing run produces only so many reads for any given sample), a change in the relative frequency for some genes will be accompanied by a change in the opposite direction in the relative frequency of reads for other genes (Table 1). This compensatory change may cause the statistical test to identify other genes as differentially expressed when in fact they are unchanged in their expression. We posit that for the large majority of cases this issue is negligible because the changes in relative frequency will be relatively small and randomly distributed through a substantial number of non-differentially expressed genes. However, problems can be envisioned for cases such as overexpression studies or in characterization of mutant genes with strong pleiotropic effects on gene expression. Methods have been proposed that effectively adjust the library sizes by some normalization factors based on the assumption that the majority of genes are not differentially expressed between different treatment groups [55,56].

**Table 1:** Potential effect of relative frequency on differential expression.

**Gene name**	**Relative frequency**					

	Sample1.1 *	Sample1.2 *	Sample1.3*	**Sample2.1 †**	**Sample2.2†**	**Sample2.3†**
**Gene 1** **§**	**11**	**13**	**14**	**55**	**52**	**57**
Gene 2	5	4	7	1	0	0
Gene 3	15	20	25	7	10	9
Gene 4	35	37	28	15	19	16
Gene 5	34	26	26	22	19	18
Total	100	100	100	100	100	100

* Samples1.1-1.3 represent biological replicates from treatment group 1.

† Samples2.1-2.3 represent biological replicates from treatment group 2.

§ Gene 1 is differentially induced in treatment group 2 relative to treatment group 1. With the fixed library size, such as an arbitrary number of 100 total reads in this example, an increase in the number of reads for gene 1 in samples 2.1–2.3 will cause compensatory decreases in the number of reads from other expressed genes 2-5 within this treatment group.

Another source of variability is the different transcript lengths present within a transcriptome. Assuming comparable expression levels, genes that encode longer transcripts are expected to produce more fragments and consequently have more assigned RNA-Seq reads than those with shorter transcripts. The longer genes will therefore appear to be more abundantly expressed than comparably expressed shorter genes. Hence, one solution is to normalize per arbitrary number of bases [20,53,57]. This approach has the potential to be misleading when the length of a transcriptional unit is poorly defined, which is the case for bacterial genes belonging to polycistronic operons. Analysis of RNA-Seq derived from host-associated bacteria indicates that a significant number of genes are encoded as operons and that nearly half of the operons display a step-wise decrease in expression [28,39]. The high number of genes expressed from polycistronic operons is supported by computational predictions in bacterial genomes [58]. As such, unless reads are equally distributed, normalization for transcript length may result in under- and overweighting of a fair number of genes unknowingly contained within an operon. The use of RNA-Seq to first resolve transcriptional units will help to overcome this concern.

After normalization of the data, the task for identifying differentially expressed genes appears simple; it is merely to apply a statistical test for comparing two treatment groups of biologically replicated samples. For analysis of microarrays, this is straightforward because the assumptions of the two-sample *t*-test are met after intensity values are log transformed. This is not the case for RNA-Seq data because the comparison is based on groups of read counts and their probability distribution cannot be approximated by a normal distribution, even after transformation. Our studies using simulated data have shown that *t*-tests are greatly underpowered and will give an unacceptably high false negative rate [59]. In other words, many truly differentially expressed genes would be missed. Thus, the tools developed for analysis of microarrays do not appear appropriate for analysis of RNA-Seq data.

The Poisson probability distribution is a natural alternative to the normal for read count data. However, the inappropriateness of the Poisson distribution for RNA-Seq data has been repeatedly demonstrated [48,56,59]. The reason is a phenomenon called overdispersion where the observed inter-library variability is substantially greater than that predicted by the Poisson model. Because of overdispersion, the variability between groups, including variability between biological replicates, will cause a Poisson test to have an actual false discovery rate substantially greater than the nominal rate [59].

When choosing a statistics package for data analysis, the appropriateness of the method in addressing small sample size and overdispersion should therefore be considered. Several packages are available, including the updated version of Cuffdiff from the Cufflinks suite of tools, edgeR, DESeq, NBPSeq, Myrna, and LOX (http://cufflinks.cbcb.umd.edu/, [48,56,59–62]). The first four packages use the negative binomial (NB) probability distribution because the NB offers a richer model for count variability. The NB distribution can be considered as a gamma mixture of Poisson distributions. In other words, the Poisson distribution explains the technical variability and the gamma distribution explains the variability between biological replicates [63]. Another important aspect is that the NB distribution permits an exact test for two-group comparisons, which means that it does not rely on large sample size asymptotic theory. For example, the DESeq package was used to analyze an RNA-Seq experiment with only two biological replicates of host-infected *Vibrio cholerae* and identified all known key virulence factors as differentially expressed [26].

There are, however, two practical issues with the use of a NB test. The first is the pooling of information from different genes to estimate the NB “dispersion parameter”, an additional parameter for variation that circumvents the main flaw in Poisson tests. Pooling has an important benefit in providing a higher true discovery rate of differentially expressing genes, *i.e.*, substantially more power in detecting truly differentially expressed genes. For small sample sizes, the power of the NB test would be substantially greater if the dispersion parameter were known, rather than estimated from the data because much of the information in the data used to compare the means will be sacrificed by the need to estimate the dispersion parameter. Of course, there is no way around the fact that the dispersion parameter is unknown but loss in power can be avoided if commonality in the dispersion parameter across genes can be exploited. For example, in a simple case, the dispersion parameter is the same for all genes and a single estimate can be obtained by pooling the information from all genes. Although each gene would contribute a very small bit of information about the dispersion parameter, the result of pooling from thousands of genes is an estimate that can be essentially treated as known.

In the original edgeR statistics package, the dispersion parameter was indeed assumed to be constant for all genes [61]. While this assumption may hold true for Serial Analysis of Gene Expression (SAGE) data, which was its original intended application, it does not appear to be the case for RNA-Seq data [56,59]. Henceforth, alternative methods were developed that are intermediate to assuming a constant dispersion parameter for all genes and separate dispersion parameters for each gene. The “moderated dispersion” version of the edgeR package uses an empirical Bayes approach, or inference based on the data, to shrink each gene's dispersion estimates towards a constant value. The “trend option” of edgeR allows the genes' dispersion estimates to vary around a nonparametric smooth curved function of the mean instead of a constant value. In the DESeq statistics package, the dispersion parameter is modeled as a nonparametric smooth function of the mean [56]. The most recent updates to the suite of tools of the Cufflinks package include a similar approach as the DESeq method [64]. Finally, in the NBPSeq statistics package, the dispersion parameter is modeled as a simple parametric function of the mean [59].

The second issue with using the NB test is that the mathematical derivation of the exact test requires library sizes to be the same, or at least approximately equal, for all biological samples. Technically, this is a nearly impossible task as several variables beyond the control of the experimental biologist contribute to producing different numbers of reads for each sample preparation. Thus, implementation of the test requires an adjustment to read counts on a scale in which library sizes are equal. The different packages differ slightly in the methods used to adjust library sizes.

**Figure 3:**
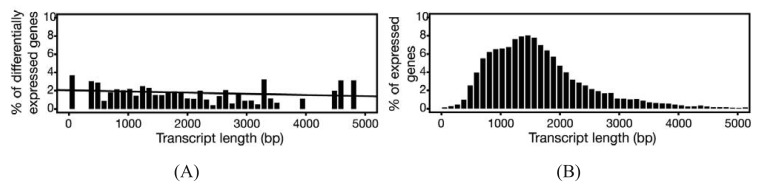
Differential expression as a function of transcript length. RNA-Seq data of transcriptomes from *Arabidopsis thaliana* infected with nonpathogenic bacteria or mock inoculated were analyzed using the GENE-counter pipeline configured with the NBPSeq package. (**A**) The differentially induced genes (y-axis) were binned based on equal range of transcript lengths (x-axis). A regression line is plotted. (**B**) Expressed genes from all replicates from both treatments are represented as a percentage within each bin defined based on equal range of transcript length.

In experiments where gene expression is being compared between treatment groups, the variability due to differences in transcript lengths and other technical biases that we have not discussed, are less of an issue, since they presumably affect the same genes to the same degree across different treatment groups. The same cannot be said for other types of analyses that rely on direct or indirect comparisons of expression of a set of genes, such as network or pathway analyses, systems studies, and analysis for enriched gene ontology (GO) terms. Since tests for differential expression are usually more powerful for genes encoding longer transcripts, tests for sets of enriched and differentially expressed genes may be biased towards those that are on average longer in length [65]. To address this issue, a weighted sampling method has been proposed to compensate for length differences [66]. We note, however, that in the original study, the problem of overdispersion was not well understood and some of the data examples that were characterized did not include biological replicates [65]. When we used NBPSeq to identify differentially induced genes from an RNA-Seq dataset comparing transcriptome changes of a host plant challenged with bacteria versus a mock inoculation, we did not observe substantial correlations between differential expression and transcript length (Figure 3) [67]. We feel that further study is needed to fully appreciate the scope and severity of this so-called “length-bias” issue.

## 5. Conclusions: RNA-Seq Has Yet to Peak

The use of RNA-Seq to investigate transcriptomes of host-associated bacteria has yielded great insights into their complexity and will do the same to help address our knowledge gap in understanding the lifestyles of plant pathogenic bacteria. Collaborative teams with plant pathologists, computer scientists, and statisticians are essential. There is a need to develop systematic and unbiased approaches for RNA-Seq to help discover genes, refine transcriptional start sites, clarify operon structures, resolve nested genes, and identify differentially expressed genes. Also necessary are new tools for integrating and visualizing large -omic datasets to help biologists formulate hypothesis. There is an urgent demand for statistical methods applicable to more complex experimental designs for RNA-Seq that involve multiple variables such as genotypes of both host and pathogen, communities of bacteria, time after infection, *etc.* The currently available exact test based on the NB distribution, while more powerful than large sample tests, apply only to two-group comparisons and does not easily extend to the regression setting necessary for characterizing RNA-Seq experiments beyond the simple two-group comparison.

For the plant pathologists, RNA-Seq can be used in combination with ChIP-seq (Chromatin immunoprecipitation coupled with next gen sequencing) and genetic mutants to help define regulons of transcriptional regulators [68]. There will be a great gain in using RNA-Seq to study economically important, but perhaps “non-model” pathogens of food crops. RNA-Seq also has potential use in studying plant pathogens during biologically relevant interactions with their hosts [69]. Thus far, studies of bacteria associated with their hosts have relied on bacterial enrichment to help with subsequent steps of enriching for bacterial RNA [26,32,51,70]. The half-life of prokaryotic RNAs is very short, usually only a number of minutes long. In *E. coli*, for example, total mRNA is estimated to have a half-life of only 6.8 minutes [71]. Thus, the more time-consuming the bacterial purification step, the more likely that host-dependent transcriptome changes will be diminished and conclusions will be biased towards genes with more stable transcripts. To adequately capture biologically interesting transcripts, bacterial enrichment methods require an early step to stabilize RNA that does not cause excessive liberation of RNA from the host.

Another challenge is that, during certain life stages, the low densities of plant pathogenic bacteria may yield insufficient quantities of RNA for sequencing. Even at high densities in culture, there may be transcriptional heterogeneity within a clonal, synchronized population [72]. A transcriptomic-based investigation of single cells is technically possible, as the transcriptome of a single bacterial cell, captured using laser microdissection and amplified using rolling circle amplification with φ29 DNA polymerase, can yield sufficient quantities of RNA for use in analysis of microarrays [73]. Additional studies have suggested that this method could apply to RNA-Seq, though it has not been explicitly tested.

*P. syringae* has seeded a change to RNA-Seq-based inquiries of plant pathogens [25]. This is befitting, since in addition to being an important model plant pathogen, *P. syringae* is hypothesized to seed clouds, an interesting but challenging niche for an RNA-Seq experiment [74]. Find a cloud, subscribe to a cloud, and start sequencing.
